# MDM2 Inhibition in the Treatment of Glioblastoma: From Concept to Clinical Investigation

**DOI:** 10.3390/biomedicines11071879

**Published:** 2023-07-02

**Authors:** Karolina I. Pellot Ortiz, Julian S. Rechberger, Leo F. Nonnenbroich, David J. Daniels, Jann N. Sarkaria

**Affiliations:** 1 Department of Radiation Oncology, Mayo Clinic, Rochester, MN 55905, USA; pellotortiz.karolina@mayo.edu; 2 Department of Neurologic Surgery, Mayo Clinic, Rochester, MN 55905, USA; rechberger.julian@mayo.edu (J.S.R.); leo.nonnenbroich@kitz-heidelberg.de (L.F.N.); daniels.david@mayo.edu (D.J.D.); 3 Department of Molecular Pharmacology and Experimental Therapeutics, Mayo Clinic Graduate School of Biomedical Sciences, Rochester, MN 55905, USA; 4 Hopp Children’s Cancer Center Heidelberg (KiTZ), 69120 Heidelberg, Germany; 5 Clinical Cooperation Unit Pediatric Oncology, German Cancer Research Center (DKFZ) and German Consortium for Translational Cancer Research (DKTK), 69120 Heidelberg, Germany

**Keywords:** glioblastoma, MDM2, p53, targeted therapy, small molecule, clinical trial, navtemadlin, idasanutlin, ALRN-6924, BI-907828

## Abstract

Inhibition of the interaction between MDM2 and p53 has emerged as a promising strategy for combating cancer, including the treatment of glioblastoma (GBM). Numerous MDM2 inhibitors have been developed and are currently undergoing rigorous testing for their potential in GBM therapy. Encouraging results from studies conducted in cell culture and animal models suggest that MDM2 inhibitors could effectively treat a specific subset of GBM patients with wild-type *TP53* or functional p53. Combination therapy with clinically established treatment modalities such as radiation and chemotherapy offers the potential to achieve a more profound therapeutic response. Furthermore, an increasing array of other molecularly targeted therapies are being explored in combination with MDM2 inhibitors to increase the effects of individual treatments. While some MDM2 inhibitors have progressed to early phase clinical trials in GBM, their efficacy, alone and in combination, is yet to be confirmed. In this article, we present an overview of MDM2 inhibitors currently under preclinical and clinical investigation, with a specific focus on the drugs being assessed in ongoing clinical trials for GBM patients.

## 1. Introduction

Among primary brain and other central nervous system (CNS) tumors, IDH-wildtype glioblastoma (GBM) is the most common and aggressive form associated with an abysmal prognosis. Approximately 13,000 cases of GBM are diagnosed per year in the United States, comprising 14% of all brain tumors and 50% of all malignant brain tumors [1]. GBM is slightly more common in males, with a male-to-female ratio of approximately 1.6 and is most often diagnosed in non-Hispanic whites [2,3]. These tumors most often develop de novo and can affect any regions within the CNS, although they most commonly affect the cerebral hemispheres, particularly the frontal and temporal lobes [4]. GBM is more common in older adults with a median age at diagnosis of 65 years. The incidence increases with age, peaking in individuals aged 75 to 84 years [1]. Conversely, GBM is less common in children, comprising less than 3% of all CNS tumors reported among the ages of 0–19 years [5].

Significant progress has been made in the last decade in developing effective precision medicine strategies for many malignancies, while minimal progress has been made for GBM. Treatment of GBM typically involves surgical resection to the extent feasible, followed by radiation therapy (RT) with concurrent and adjuvant and cytotoxic chemotherapy [6]. The last FDA-approved drug for newly diagnosed GBM was temozolomide (TMZ), which extended the median survival by only 2 months [6,7]. Although a number of variables have been linked to TMZ resistance, expression of O6-methylguanine-DNA-methytransferase (MGMT), which removes TMZ-induced cytotoxic O6-methylguanine lesions, remains an important contributor [8]. Expression of MGMT is regulated by promoter methylation, and the ~40% of patients with GBM promoter hypermethylation have a much longer survival (median 23 months) as compared to unmethylated GBM patients (14 months) [9]. Regardless of MGMT status, the 5 year survival for GBM patients is less than 5% [1,4]. Consequently, there is a compelling unmet need to develop novel effective therapies for GBM.

Seminal studies over the past decades have revolutionized our understanding of the molecular basis of this disease and facilitated the identification of novel targets in tumor cells and the tumor microenvironment potentially amenable to therapeutic intervention [10]. Molecularly targeted agents that target signaling pathways critical for tumor cell growth, invasion, proliferation, apoptosis, and DNA repair have been extensively evaluated for therapy in GBM [11]. Here, we focus our discussion on the interaction between the tumor suppressor p53 and the E3 ubiquitin ligase murine double minute 2 (MDM2) and how this molecular interplay may be exploited using small molecule inhibitors. We provide an overview of MDM2 inhibitors under preclinical and clinical investigation, with a focus on drugs being evaluated in ongoing clinical trials for GBM patients.

## 2. Basic Biology

The p53 signaling pathway is a critical mediator of cell fate following genotoxic damage and provides key tumor suppressor functions. The central player in this signaling network, p53, is encoded by *TP53*, and upon cellular stress and DNA damage, it is simultaneously activated and stabilized inside the cell [12,13]. Under such circumstances, p53 functions as a transcriptional activator, binding to the promoters of hundreds of genes, including those crucial for genotoxic stress recovery, initiation of senescence, and apoptosis activation. P53 also prompts the activation of transcriptional repressive complexes such as the dimerization partner, RB-like, E2F, and the multi-vulval class B (DREAM) complex, which silences hundreds of other genes, including those essential for cell cycle progression and particular elements of multiple DNA repair pathways [14,15,16,17,18,19,20,21,22]. This coordinated DNA damage response enables the restoration or removal of damaged cells that may be tumorigenic [23,24]. Since high p53 levels can be detrimental to the growth and development of normal cells, intracellular p53 levels are tightly regulated by ubiquitination and rapid proteasomal degradation as well as other mechanisms under non-stressed conditions [25,26].

*TP53* is the most frequently mutated gene across all types of cancer, with mutations occurring in about 50% of malignancies [24,27,28]. The mutation prevalence of *TP53* among different cancer types varies, ranging from less than 5% in cervical cancer to up to 90% in ovarian cancer and small cell lung cancer [24]. In lower grade (CNS WHO grade 2 and 3) gliomas, *TP53* is deregulated in approximately 50% of cases, most commonly in co-occurrence with *IDH1* mutations [29,30,31]. The majority (>70%) of CNS WHO grade 4 IDH-mutant astrocytomas harbor *TP53* mutations [32,33,34]. According to The Cancer Genome Atlas Program (TCGA), *TP53* is mutated in up to 30% of GBMs [28,35]. While missense substitutions account for the most common *TP53* mutations (~70%), other alterations, such as frameshift insertions/deletions, nonsense mutations, and silent mutations, are less common [24]. Human cancers frequently have segmental deletions on chromosome 17p, which involves the *TP53* locus [36]. Although most *TP53* mutations are detrimental (i.e., they cause a loss of function), some mutations in *TP53* are innocuous, allowing p53 to continue acting as a transcription factor [37]. There have also been reports of tumorigenic gain-of-function mutations in *TP53* [27]. Given the pivotal role of p53 in the response to genotoxic stress and the prevalent dysregulation of p53 in almost all types of cancer, there is intense interest in harnessing potential therapeutic vulnerabilities to restore or hyper-activate p53’s function.

**Figure 1:**
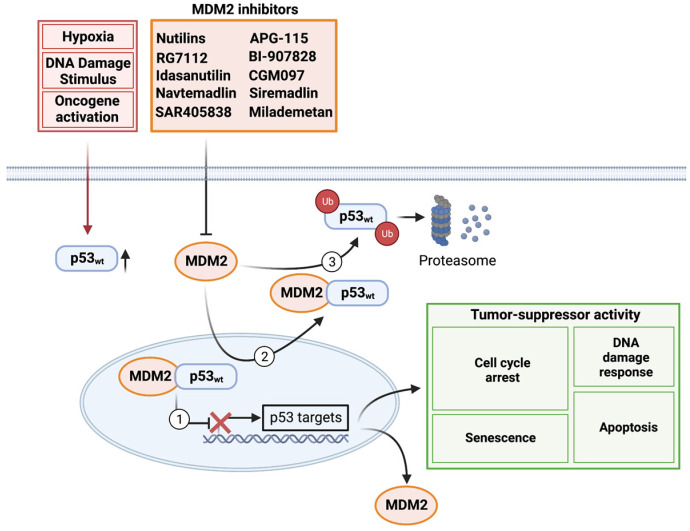
Summary of the p53–MDM2 regulatory loop and its intracellular implications. (1) MDM2 prevents p53 from regulating the transcription of MDM2 and other downstream target genes by binding to p53 directly. (2) Through interaction with various cytoplasmic proteins, MDM2 promotes SUMOylation and nuclear export of p53. (3) Most notably, MDM2 is an E3 ubiquitin ligase that targets p53 via ubiquitination for proteasomal degradation and thereby suppresses p53 transcriptional activity. All of these mechanisms work together to reduce p53 activity in the absence of cellular stress. Created with BioRender.com (accessed on 15 February 2023).

While p53 is continuously expressed, protein levels and, consequently, the transcriptional activity of p53 are normally suppressed by MDM2 (Figure 1). MDM2 is an E3 ubiquitin ligase that targets p53 via ubiquitination for degradation by the proteasome [38]. Through its interaction with PIASy, a SUMO E3 ligase, MDM2 also promotes SUMOylation and nuclear export of p53 [39]. The interplay of p53 and MDM2 creates an autoregulatory feedback loop, whereby p53 binds to the P2 promoter of *MDM2*, ultimately increasing MDM2 transcript and protein levels. By binding to p53 directly (as well as by blocking the p53 transactivation domain), MDM2 then prevents p53 from mediating the transcription of *MDM2* and other downstream target genes [25,39,40,41]. Collectively, these mechanisms function to suppress p53 activity in the absence of cellular stress.

MDM2 is upregulated in a multitude of cancers and is associated with a poor prognosis [42]. Gene amplification, single-nucleotide polymorphisms in the promoter region, increased transcription, and enhanced translation of *MDM2* may cause an enhanced degradation and decreased activity of p53 [43,44]. *MDM2* amplification is the most frequent cause of MDM2 overexpression and occurs in approximately 3.5% of all malignancies [45]. Up to 65% of well-differentiated and de-differentiated liposarcomas have a high rate of *MDM2* amplification, while several other solid malignancies, including GBM, soft-tissue and bone sarcomas, gallbladder and duodenal adenocarcinoma, lung adenosquamous carcinoma, and ovarian carcinosarcoma, have a frequency of approximately 15% [28,45]. In most tumors, *MDM2* amplification is mutually exclusive to *TP53* mutation. Consistent with *MDM2* amplification being a truncal, driver oncogenic event that functionally inactivates p53, *MDM2* amplification is highly homogeneous within tumors [46]. Consequently, an appealing therapeutic approach for reactivating p53 in cancers with wild-type *TP53* or functional p53 is to target the MDM2–p53 interaction. Instead of targeting an oncogenic driver, this approach focuses on activating a tumor suppressor, and, as such, requires functional p53 to be present in targeted cells.

## 3. Preclinical Evaluation and Clinical Development of MDM2 Inhibitors

Small molecule inhibition blocking the MDM2–p53 interaction is the most clinically advanced therapeutic strategy for restoring p53 function. Crystallographic analyses of MDM2 bound to p53 have provided the framework for the identification of numerous structurally distinct small molecule disruptors of this interaction [47]. Consistent with the signaling described in the preceding section, MDM2 inhibitors cause pronounced accumulation of p53 and a robust upregulation of p53-mediated transcription in tumors with wild-type *TP53*, regardless of the *MDM2* amplification status [48,49,50]. Conversely, *TP53*-mutant or *TP53*-null cells are resistant to MDM2 inhibitors as the accumulation of functional p53 is mechanistically linked to the anti-tumor effects [51,52]. Numerous MDM2 inhibitors are in clinical development for a variety of cancers including GBM (e.g., idasanutlin, navtemadlin, APG-115, BI-907828, CGM097, siremadlin, and milademetan), and current clinical experience suggests these drugs can effectively modulate p53 accumulation and transcriptional activity in human tumors with tolerable normal tissue toxicities [47,53,54]. Published results from Phase 1 studies with these agents demonstrate promising activity in acute myeloid leukemia (AML), polycythemia vera, and sarcoma with presumed on-target, dose-limiting gastrointestinal and hematologic toxicities [47,55]. Early phase, GBM-specific trials with navtemadlin, idasanutlin, and BI-907828 are currently ongoing. Below is a description of salient pre-clinical and clinical data across a spectrum of MDM2 inhibitors in clinical development for GBM.

### 3.1. Nutilins

Nutlins (nutlin-1, -2, and -3) were the first selective and potent molecules reported to interrupt the p53–MDM2 interaction [56,57,58]. The original proof-of-concept nutlin, nutlin-3a, displayed activity in p53 wild-type cells but not in p53 defective cells [56]. In additional preclinical investigations, nutlin-3a was active in leukemia and other cancer types with intact p53 function [57,59]. Nutlin-3a treatment of glioma cell lines and primary grown GBM cells demonstrated p53-dependent G1- and G2-M cell cycle arrest and apoptosis specifically in p53 wild-type cells [47,60]. Furthermore, nutlin-arrested glioma cells exhibited structural signs of senescence and a sustained p21 protein increase [60]. The fact that GBM cells with targeted siRNA suppression of *TP53* or cells with mutant or functionally impaired p53 were fully unresponsive to nutlin-3a suggested that nutlin-3a-induced apoptosis and senescence are reliant on an intact p53 function [47,60].

### 3.2. RG7112 (RO5045337) and Idasanutlin (RG7388)

Similar to nutlin-3a, the nutilin derivative RG7112 (RO5045337) is a nongenotoxic inhibitor that blocks the p53–MDM2 interaction by binding to the p53 pocket on MDM2 [56,61]. RG7112 results in p53 accumulation and activation of p53 signaling in cancer cells that express wild-type p53, which has been shown to deplete MDM2-upregulated progenitor/stem cells and inhibit the growth of human tumor xenografts by causing cell cycle arrest and apoptosis [62,63]. Preliminary clinical data suggest efficacy in solid tumors [64] and hematologic malignancies [63,65]; however, this compound is not currently being pursued in clinical trials.

Idasanutlin (RG7388) shares the same cellular mechanism as RG7112, but it affords improved selectivity, potency, and bioavailability over the predecessor RG7112 [58,66]. Idasanutlin showed strong anti-cancer efficacy in preclinical studies in p53 wild-type xenograft tumor models. In vitro and in vivo, RG7388 induces cell cycle arrest and death at 10-fold lower doses than RG7112 [66]. One study demonstrated that exposure to idasanutlin in p53 wild-type GBM led to a dose-dependent reduction in clonogenicity and proliferation [67]. Results from preclinical studies demonstrate the potential for idasanutlin as a potential therapy for solid and hematologic malignancies, and the drug is now undergoing clinical testing [68,69]. Several clinical trials are ongoing, including a Phase 1/2a study in GBM patients without MGMT promoter methylation (NCT03158389). This trial is investigating a number of different targeted compounds in combination with RT based on molecular characterization and is expected to be completed in September 2024.

### 3.3. Navtemadlin (KRT-232/AMG-232)

Navtemadlin (KRT-232/AMG-232) provides further improvements in both biochemical and cellular efficacy in comparison to earlier nutlin MDM2 inhibitors [70]. In preclinical studies of several cancers, navtemadlin strongly increased p53 activity, arresting the cell cycle and reducing the growth of tested tumor models [71,72]. A strong association between navtemadlin effectiveness and intact p53 function was reported in patient-derived GBM cells. Navtemadlin was found to be 9.5 times more potent than RG7112 in p53 wild-type GBM cells. The same study also demonstrated that navtemadlin sensitivity was 35-fold higher in *MDM2*-amplified stem cells, indicating that the cellular context may influence navtemadlin effectiveness [72]. Through cell proliferation arrest and apoptosis induction, navtemadlin prevented the in vivo growth of numerous tumor xenografts and caused complete and durable regression of *MDM2*-amplified SJSA-1 sarcoma tumors [71]. The drug is currently being tested in multiple clinical trials, including one for patients with recurrent GBM and patients with newly diagnosed GBM harboring unmethylated MGMT promoters and wild-type p53 (NCT03107780).

### 3.4. SAR405838 and APG-115

SAR405838 and APG-115 are structurally similar, highly potent MDM2 inhibitors that were both designed and optimized at the University of Michigan. The former was demonstrated to trigger wild-type p53, resulting in p53-dependent cell cycle arrest and cell death in preclinical models of leukemia and solid tumors, such as osteosarcoma, prostate cancer, and colon cancer [73]. *MDM2*-amplified patient-derived models of GBM were highly sensitive to SAR405838 in comparison with *MDM2* non-amplified lines in vitro and in subcutaneous flank models. Interestingly, SAR405838 was ineffective in GBM patient-derived xenografts grown in the brain of mice, which was attributed to the limited distribution across the blood–brain barrier (BBB) [74]. Two Phase 1 studies in patients with advanced solid tumors found an acceptable safety profile with SAR405838 [75,76]; however, the drug is no longer in clinical development. The same group that developed SAR405838 subsequently discovered APG-115, which possesses a better solubility profile than SAR405838 and had potent anti-cancer effects in preliminary studies of multiple tumor cell lines and xenograft models [77]. Specifically, APG-115 potently activated wild-type p53 and selectively inhibited the growth of p53 wild-type human cancer cell lines, with IC_50_ values in the low nanomolar range. In vivo, an excellent pharmacokinetic profile allowed APG-115 to effectively activate wild-type p53 in an osteosarcoma xenograft mouse model following a single oral dose, where it afforded complete tumor remission when administered at 100 mg/kg daily for 14 days. Similar effects were achieved in an acute lymphoblastic leukemia xenograft model in mice with both 100 mg/kg daily for 14 days and 200 mg/kg weekly for 3 weeks. Several Phase 1 and Phase 2 clinical trials are now recruiting patients for treatment with APG-115.

### 3.5. BI-907828

BI-907828 is a potent MDM2 inhibitor with a favorable solubility, bioavailability, and systemic clearance profile in a variety of animal models that has completed first-in-human Phase 1 testing [78,79]. It has a picomolar binding affinity for the p53–MDM2 interface and is highly potent in cell culture and xenografts for GBM and sarcoma [80,81]. In *MDM2*-amplified orthotopic xenograft mouse models of GBM, BI-907828 treatment significantly extended survival when used as monotherapy and when combined with TMZ [80]. In combination with a PD-1 checkpoint inhibitor, BI-907828 also demonstrated synergistic effects in vivo [82]. A low effective dose was anticipated in people based on preclinical model correlation studies and human pharmacokinetic prediction models [82]. Phase 1 evaluation of BI-907828 monotherapy in 89 heavily pre-treated patients (NCT03449381) demonstrated a maximally tolerated dose of 60 mg dosed orally once every 21 days, and confirmed that partial responses were observed in over a quarter of liposarcoma patients and in several other *MDM2*-amplified tumors. Based on these promising data, there are now several ongoing Phase 1, 2, and 3 clinical trials enrolling patients with a variety of solid tumors. An ongoing Phase 0/1 trial (NCT05376800) in patients with newly diagnosed GBM is measuring BI-907828 distribution and pharmacodynamic effects in different tumor regions.

### 3.6. CGM097

CGM097 is a potent and selective MDM2 inhibitor with an excellent in vivo profile currently in Phase 1 clinical development [83,84]. In multiple p53 wild-type cell lines, CGM097 demonstrated a pronounced inhibition of cell proliferation [84]. Interestingly, CGM097 was found to inhibit ABCB1-associated ATPase in an in vitro study [85], which suggests the potential of this drug to counteract multi-drug resistance in conjunction with chemotherapeutic drugs to improve anti-tumor responses. The synergistic and additive effects of CGM097 have been reported in combination with the BET inhibitor OTX015 and the antimetabolite 5-fluorouracil in neuroblastoma and neuroendocrine tumor cell lines, respectively [86,87]. CGM097 significantly prolonged survival in patient-derived xenograft rodent models of B-cell acute lymphoblastic leukemia and solid tumors with functional p53 [84,88]. The lymphoid organs, bone marrow, GI tract, and testes were identified as the target organs in toxicology experiments on monkeys [84]. A Phase 1 dose escalation clinical trial in adult patients with advanced solid tumors has been completed, but the results have not been reported.

### 3.7. Siremadlin (HDM201)

Characterization and chemical optimization of CGM097 facilitated the development of siremadlin (HDM201) as a clinical candidate to inhibit the p53–MDM2 interaction. Preclinical studies demonstrated siremadlin upregulates p53 activity both in vitro and in vivo [89,90]. Siremadlin was highly potent and selective for p53 wild-type cells when tested in a large panel of cancer cell lines [91]. In tumor-bearing rodents, siremadlin displayed excellent activity with oral dosing [91,92]. Clinical development of siremadlin was recently initiated based on a promising preclinical efficacy and a favorable biochemical/biophysical profile [93]. A Phase 1 study in patients with advanced hematological and solid tumors characterized by wild-type p53 reported manageable safety and preliminary activity with siremadlin administered either in a high dose intermittent or a low dose extended regimen [94,95]. Other Phase 1 and Phase 1/2 clinical trials for siremadlin are ongoing in addition to this study.

### 3.8. Milademetan (DS-3032b)

Milademetan (DS-3032b) is a potent, selective, and orally available inhibitor of the p53–MDM2 interaction that drives p53 signaling in multiple preclinical models and has advanced to clinical testing [96,97]. The drug has promising anti-tumor activity in cell lines and xenograft models of AML, non-Hodgkin’s B-cell lymphoma, neuroblastoma, and Merkel cell carcinoma [96,98,99]. Multiple clinical trials are investigating milademetan alone and in combination therapy regimens [47]. Early phase clinical studies in patients with AML, lymphoma, advanced liposarcoma, and other solid tumors have reported a favorable safety profile with notable single-agent activity in dedifferentiated liposarcoma (CCC) [100,101,102], which has prompted the initiation of a randomized Phase 3 trial (NCT04979442).

### 3.9. ALRN-6924 (Dual MDM2/MDMX Inhibitor)

An alternative strategy to the above-described method of selectively disrupting MDM2–p53 binding is dual inhibition of the p53–MDM2/MDMX interaction [103]. Dual MDM2/X inhibitors are considered a separate class of molecules; thus, they are not discussed in detail herein. The interested reader is kindly referred to an excellent review by Lemos et al. [104], which provides an in-depth overview of this concept.

ALRN-6924 is a first-in-class dual MDM2/MDMX inhibitor with excellent on-target activity in multiple in vitro and in vivo cancer models that has completed Phase 1 clinical testing [47,105]. The drug robustly activates p53-dependent transcription in preclinical models of p53 wild-type leukemia and solid tumors. In cell lines and primary AML patient cells, including leukemic stem-cell-enriched populations, dual MDM2/MDMX inhibition by ALRN-6924 resulted in dose-dependent cycle arrest and apoptosis [106]. Furthermore, it decreases the tumor burden and markedly improves survival in AML xenografts [106]. When used with paclitaxel and eribulin on hormone receptor positive (ER+) breast cancer cells, ALRN-6924 has synergistic effects in vitro and an enhanced efficacy in vivo [107]. A first-in-human Phase 1 clinical trial in patients with solid tumors or lymphomas reported excellent tolerability and some promising signs of anti-tumor activity [105]. In light of these encouraging results, additional Phase 1 studies are currently evaluating ALRN-6924 alone or in combination with cytotoxic chemotherapy, including one in children with solid tumors, leukemia, lymphoma, and brain tumors (NCT03654716).

## 4. Combination Therapy

**Figure 2:**
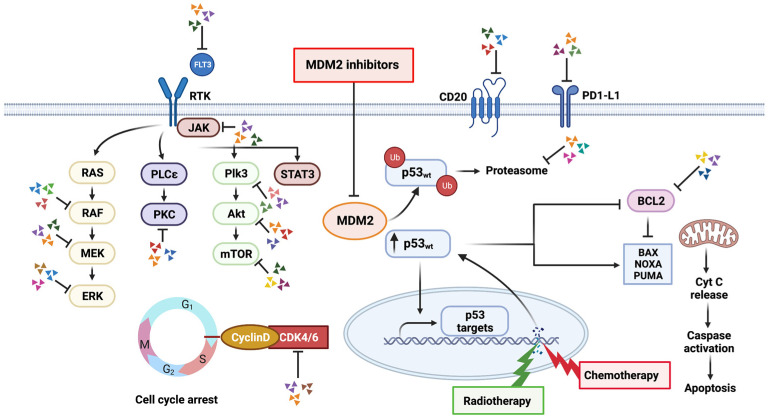
Potential synergistic interactions between different therapeutic modalities and MDM2 inhibitors. Chemotherapy and radiation therapy (RT) remain the most promising and thoroughly researched combination partners for MDM2 inhibition. MDM2 and p53 activities are closely associated with the efficacy of chemotherapeutic drugs that work by damaging DNA, and inhibition of MDM2 renders tumor cells more susceptible to these types of pharmacotherapies. MDM2 inhibitors increase the effectiveness of RT by radiosensitizing tumor cells. Additionally, potential synergistic pathway inhibition with small-molecule-targeted therapies and other treatment modalities, such as immunotherapy, is conceivable and the subject of ongoing research (colored arrowheads signify examples of targeted molecules). Created with BioRender.com (accessed on 15 February 2023).

The standard of care for newly diagnosed GBM includes surgical resection, combined RT and TMZ for six weeks, and adjuvant TMZ for six months [108,109]. Locally administered radiation kills tumor cells by inducing DNA double-strand breaks in dividing cells [108,110,111]. When the DNA in a cell is damaged, the cell may be unable to repair the damage or may fix it improperly, which can lead to cell death [112,113,114]. When cells experience DNA damage or other cellular stress, p53 levels increase, leading to various outcomes, including cell cycle arrest, DNA repair, or apoptosis [115]. When DNA damage reaches a critical point, p53 levels increase past a threshold that triggers the upregulation of pro-apoptotic genes, including Bax and PUMA, while concurrently downregulating the expression of anti-apoptotic genes, such as Bcl-2, ultimately leading to the initiation of apoptosis [116]. Chemotherapy is a commonly used cancer treatment strategy, primarily because cancer cells usually demonstrate accelerated rates of cell division and replication, making their DNA more susceptible to damage than that of normal cells [117,118,119,120]. Genotoxic chemotherapy drugs can cause different types of DNA damage, such as double-strand breaks. Such damage can disrupt the cellular ability to undergo division and eventually result in cell death [117,119,120,121]. MDM2 antagonists disrupt the p53/MDM2 interaction, resulting in accumulation of p53 [122,123,124,125,126,127]. Therefore, by combining an MDM2 inhibitor with chemotherapy or RT, higher levels of p53 can be achieved, leading to the activation of genes that lead to apoptosis (Figure 2) [115,128,129,130].

### 4.1. Radiation Therapy

Combined RT and MDM2 inhibition provides synergistic effects in cancer treatment [131,132,133]. In normal cells, p53 levels increase in response to DNA damage and repair mechanisms are initiated, or apoptosis is induced if the damage is too severe [134]. RT causes DNA damage in cancer cells, which in turn activates the p53 pathway, ultimately leading to apoptosis. In the same way, inhibiting MDM2 increases p53 levels in the cell, which can sensitize cells to RT and enhance the effectiveness of RT [47]. The synergistic effect between RT and MDM2 inhibitors can be attributed to two different triggers that drive p53 activation [135,136]. By increasing p53 levels, both treatments together cross the threshold for apoptosis rather than DNA damage repair, leading to a more efficient elimination of cancer cells.

In preclinical models of GBM, MDM2 inhibitors can reduce tumor growth, prolong survival, and enhance the cytotoxicity of RT when used in combination. One study showed that the combination of nutlin-3 and ionizing radiation resulted in apoptosis and increased the sensitivity to radiation in p53 wild-type GBM cell lines [137]. Another study investigated the impact of RG7388 and RT on p53 wild-type GBM cell lines and glioma-initiating cells [67]. The results suggest that combining RG7388 and RT could serve as a first-line treatment for tumors that are resistant to standard chemotherapy with alkylating agents. KRT-232 in combination with RT was also shown to be an effective treatment in patient-derived xenograft models of GBM, both in vitro and in vivo [138].

### 4.2. Chemotherapy

As mentioned before, compounds that increase MDM2 levels or inhibit the MDM2/p53 interactions can affect DNA break repair and cause genome instability [139]. When combined with DNA-damaging agents, these compounds can induce higher levels of cancer cell apoptosis. Genotoxic chemotherapy drugs, such as cisplatin, doxorubicin, and etoposide, work by causing DNA damage in cancer cells, which activates the p53 pathway, ultimately leading to cell cycle arrest or apoptosis [140]. MDM2 inhibitors can sensitize cancer cells to genotoxic chemotherapy by inhibiting the activity of MDM2 and increasing the levels of p53 [117,119,141,142]. This can lead to enhanced activation of the p53 pathway in response to sustained damage without repair, leading to apoptosis [56].

Several studies have investigated MDM2 inhibitors in combination with chemotherapy to treat GBM. Combined nutlin-3a and doxorubicin treatment results in the robust activation of the p53 pathway, leading to an increase in p53 activity and, consequently, cell death of GBM cells [143]. In this study, researchers examined the synergy between nutlin-3 and doxorubicin by encapsulating them in PEG-PE-based micellar nanocarriers [143]. The effectiveness of these nanocarriers was tested against U87MG cells in both 2D and 3D models, which showed increased levels of p53 in cells, induced apoptosis, and a strong synergistic cytotoxic effect. To determine if MDM2 contributes to drug resistance in glioma, one study inhibited the expression of MDM2 genetically using siRNA or chemically using the MDM2 inhibitor RG7112 in U87 and DK-MG cells [144]. The results showed that inhibiting MDM2 increased the sensitivity of glioma cells to TMZ, as evidenced by a reduction in the number of viable cells in MDM2-deficient glioma cells. Additionally, treatment with RG7112 significantly decreased the viability of glioma cells [144]. One study sought to circumvent limitations imposed by the BBB by developing a polymeric micelle that carries a MDM2/MDMX antagonist [145]. In vitro studies showed efficient inhibition of proliferation of glioma cells by activating the p53 signaling pathway. In vivo studies demonstrated the micelles inhibited tumor growth in human GBM xenograft models in mice. Most importantly, when combined with TMZ, the drug substantially increased anti-tumor efficacy against GBM in experimental animals. Another study revealed that ISA27, an experimental small molecule inhibitor of MDM2, effectively activated the p53 function and inhibited the growth of human GBM cells in vitro by inducing cell cycle arrest and apoptosis [146]. When administered in an immunoincompetent BALB/c nude mouse model with a human GBM xenograft, ISA27 activated p53 and inhibited cell proliferation, resulting in apoptosis in tumor tissue. Furthermore, when combined with TMZ, ISA27 resulted in a synergistic inhibitory effect on GBM cell viability in vitro. When used together, TMZ/nutlin3a have also shown a synergistic effect in reducing the growth of wild-type p53 GBM cells. This effect was linked to activation of the p53 pathway, downregulation of DNA repair proteins, persistence of DNA damage, and decreased invasion [147].

### 4.3. Potential Side Effects and Toxicity Profile

While the promising results of several preclinical studies provide a rationale for further investigating the combination of clinically established treatment options such as genotoxic chemotherapy with MDM2 inhibitors in GBM patients, there are also limitations to such combinatorial approaches. Clinical trials have reported hematological toxicities with MDM2 inhibitor monotherapy, requiring careful management and patient monitoring, particularly when used in combination with drugs that have overlapping toxicity profiles [125]. Thrombocytopenia, a side effect shared by MDM2 inhibitors and clinically approved drugs for GBM (TMZ, lomustine (CCNU)), poses a challenge for the integration of these agents with MDM2 inhibitor-based regimens [148]. Other shared toxicities include metabolic abnormalities, fatigue, and cardiovascular effects, which can become more prominent when multiple drugs are used in combination. The potential for drug–drug interactions must, consequently, be considered when combining MDM2 inhibitors with other agents [59]. In this context, precision medicine strategies focused on matching the molecular features of individual patient tumors with the most effective combinatorial regimen (with non-overlapping CNS-related and systemic toxicity profiles) will be instrumental both for maximizing tumor control and limiting toxicities for individual patients.

### 4.4. Targeted Therapy

Targeted therapies that inhibit specific oncogenic mutations or signaling pathways, such as EGFR or MEK inhibitors, are being explored in combination with MDM2 inhibitors for GBM treatment [76,149,150]. One study showed that FC85 (an AKT/mTOR inhibitor) in combination with MDM2 inhibition therapy produced a synergistic effect on inhibiting cell viability and reactivating the p53 pathway [151]. Additionally, this drug combination was able to block in vitro tumor cell proliferation and promote apoptosis. Another study in a p53 wild-type GBM orthotopic mouse model showed that treatment with RG7388 in combination with trametinib restored sensitivity to RG7388 and reduced tumor growth in vivo [67]. Furthermore, combining trametinib with MDM2 inhibitors increased the effects of both treatments by attacking multiple pathways, making it a promising dual-target approach while minimizing potential side effects [67].

## 5. Clinical Trials

Extensive preclinical research has been conducted with MDM2 inhibitors and has demonstrated varying efficacy rates in vitro and in vivo in multiple experimental tumor models. Clinical studies have been set up to evaluate them as a monotherapy or in combination with conventional chemotherapy or innovative therapeutic protocols in different types of solid (e.g., breast, colon, pancreas, sarcomas, and lymphomas) and non-solid (e.g., leukemia) tumors. Preliminary findings have been published, providing data on these therapeutic techniques’ effectiveness, safety, and acceptability and the potential adverse effects they may cause. Currently, 28 clinical trials are evaluating the possible therapeutic use of MDM2 inhibitors in cancer. Of these, only a few have proceeded to Phase 3 trials, indicating the early stages of development for such therapy. Four of these trials include brain tumor patients (Table 1), but only two active studies are specifically focusing on MDM2 inhibitors for GBM.

### 5.1. NCT01723020

Kartos Therapeutics, Inc. is conducting a Phase 1 study with the goal of evaluating the potential of AMG 232 as a treatment option for patients with *TP53* wild-type advanced solid tumors or multiple myeloma (MM). The study assessed the safety, pharmacokinetics (PK), pharmacodynamics, and efficacy. To date, it has enrolled 107 participants, including 10 GBM patients. The study found that AMG 232 was safe up to 240 mg, with thrombocytopenia and neutropenia reported as dose-limiting toxicities. AMG 232 showed acceptable safety and dose-proportional pharmacokinetics and stable disease was observed in a subset of patients.

### 5.2. NCT03107780

The National Cancer Institute (NCI) is sponsoring a Phase 1 dose-escalation clinical trial to investigate the safety and tolerability of navtemadlin (KRT232/AMG232) as a monotherapy in recurrent GBM and in combination with RT for newly diagnosed GBM patients with an unmethylated MGMT promoter. This study includes a surgical window of opportunity study to determine the concentration of navtemadlin achievable in GBM tissues. The surgical sampling portion of the trial has completed accrual, and the dose escalation portion of the trial is currently suspended for a planned interim analysis.

### 5.3. NCT03158389

The University Hospital Heidelberg is sponsoring a Phase 1/2a clinical trial in patients with newly diagnosed GBM lacking MGMT promoter methylation. This clinical trial is being conducted to investigate the effectiveness of targeted therapies matched to the molecular characteristics of patients in combination with RT. The study has an estimated enrollment of 350 participants. Patients will be treated with a combination of RT and idasanutlin (RG7388). In other arms, RT is combined with other targeted drugs (APG101, alectinib, atezolizumab, vismodegib, temsirolimus, or palbociclib). The trial aims to increase the overall survival of GBM patients with an unmethylated MGMT promoter through molecular analysis and tailored drug use in a contemporary study design.

### 5.4. NCT03654716

The Dana–Farber Cancer Institute is sponsoring a clinical study to investigate the MDM2 inhibitor ALRN-6924 as a monotherapy for different types of cancer, including leukemia, brain tumors, solid tumors, and lymphoma. The trial is also investigating using ALRN-6924 combined with cytarabine to treat leukemia patients. The study is currently enrolling patients with a target accrual of 69.

### 5.5. NCT05376800

Boehringer Ingelheim is sponsoring a Phase 0/1a clinical trial open at Mayo Clinic. Patients with a clinical diagnosis of newly diagnosed GBM are eligible to participate in the surgical window of opportunity portion of the study to define achievable concentrations of BI-907828 in different tumor regions. Patients with confirmed GBM and *TP53* wild-type status after resection then may enroll in the Phase 1a portion of the study evaluating the tolerability of BI-907828 combined with concurrent RT. The trial is actively recruiting with an overall accrual goal of 35 patients.

### 5.6. Phase 3 Trials in Other Cancers

As mentioned above, a limited number of Phase 3 clinical trials with MDM2 inhibitors have been performed for cancers outside the CNS. A randomized, multicenter, open-label, Phase 3 trial evaluating the safety and efficacy of milademetan (DS-3032b) versus the anti-tumor chemotherapy trabectedin is ongoing (NCT04979442). Patients with advanced unresectable and/or metastatic dedifferentiated liposarcoma who have progressed on at least one prior systemic therapy, including at least one anthracycline-based therapy, are eligible to participate in this trial. Brightline-1 (NCT05218499) is an ongoing randomized, multicenter, open-label, Phase 2/3 trial of BI-907828 evaluating BI-907828 in comparison to doxorubicin as a first-line treatment for dedifferentiated liposarcoma [157]. This study is actively recruiting with an overall accrual goal of 390 patients. The so-called MIRROS study (NCT02545283) is another Phase 3 trial, which evaluated the safety and efficacy of idasanutlin in combination with cytarabine in patients with relapsed or refractory AML [158]. A total of 436 patients were enrolled at the time of primary analysis, including 355 in the *TP53* wild-type intention-to-treat population. Despite an improved overall response rate, adding idasanutlin to cytarabine neither improved the overall survival nor the complete remission rates in the tested patient population [159]. A Phase 2/3 trial of navtemadlin as maintenance treatment for patients with advanced or recurrent p53 wild-type endometrial cancer who have achieved complete or partial response on chemotherapy is estimated to begin enrolling patients in July of 2023 (NCT05797831).

## 6. Conclusions

Several MDM2 inhibitors have been developed and are currently being explored for their possible use in cancer treatment. Among the most extensively studied MDM2 inhibitors are nutlins, idasanutlin, navtemadlin, APG-115, BI-907828, CGM097, siremadlin, and milademetan. Studies conducted in cell cultures and animals have shown encouraging results, suggesting that MDM2 inhibitors could be effective in treating a subset of GBM patients. Some of these inhibitors have progressed to clinical testing, but their efficacy has yet to be confirmed. Further research is needed to determine the efficacy of MDM2 inhibitors in treating GBM and to identify the patient population that would benefit the most from this therapeutic approach. As the Phase 1 and 2 clinical trial evaluations mature, results from the follow-on definitive Phase 3 randomized studies will be eagerly awaited to understand the potential clinical benefit from this class of small molecule inhibitors.

## Figures and Tables

**Table 1:** Clinical trials involving MDM2 inhibitors in glioblastoma *.

NCT Number	Title	MDM2 Inhibitor	Status	Condition	Ref.
NCT01723020	A Phase 1 Study Evaluating AMG 232 in Advanced Solid Tumors or Multiple Myeloma	AMG 232	Completed	Advanced Solid Tumors Glioblastoma Multiple Myeloma	[152]
NCT03107780	Testing the Ability of AMG 232 (KRT 232) to Infiltrate the Tumor in Patients With Brain Cancer	Navtemadlin (KRT-232/AMG-232)	Suspended	Glioblastoma Gliosarcoma MGMT-Unmethylated Glioblastoma Recurrent Glioblastoma	[153]
NCT03158389	NCT Neuro Master Match—N^2^M^2^ (NOA-20) (N^2^M^2^)	Idasanutlin (RG7388)	Recruiting	Glioblastoma, Adult	[154]
NCT03654716	Phase 1 Study of the Dual MDM2/MDMX Inhibitor ALRN-6924 in Pediatric Cancer	ALRN-6924	Recruiting	Leukemia Brain Tumor Solid Tumor Lymphoma	[155]
NCT05376800	A Study to Determine How BI 907828 is Taken up in the Tumor and to Determine the Highest Dose of BI 907828 That Could be Tolerated in Combination With Radiation Therapy in People With a Brain Tumor Called Glioblastoma	BI 907828	Recruiting	Glioblastoma	[156]

* All data concerning clinical trials were obtained from ClinicalTrials.gov using “glioblastoma”, “GBM”, “MDM2”, and “MDM2 inhibitor” search terms for Condition or disease and Intervention/Treatment, respectively (accessed on 1 March 2023).

## Data Availability

No new data were created or analyzed in this study. Data sharing is not applicable to this article.

## References

[1] Ostrom Q.T., Cioffi G., Waite K., Kruchko C., Barnholtz-Sloan J.S. (2021). CBTRUS Statistical Report: Primary Brain and Other Central Nervous System Tumors Diagnosed in the United States in 2014–2018. Neuro Oncol..

[2] Carrano A., Juarez J.J., Incontri D., Ibarra A., Guerrero Cazares H. (2021). Sex-Specific Differences in Glioblastoma. Cells.

[3] Ostrom Q.T., Cote D.J., Ascha M., Kruchko C., Barnholtz-Sloan J.S. (2018). Adult Glioma Incidence and Survival by Race or Ethnicity in the United States From 2000 to 2014. JAMA Oncol..

[4] Fyllingen E.H., Bø L.E., Reinertsen I., Jakola A.S., Sagberg L.M., Berntsen E.M., Salvesen Ø., Solheim O. (2021). Survival of glioblastoma in relation to tumor location: A statistical tumor atlas of a population-based cohort. Acta Neurochir..

[5] Ostrom Q.T., Price M., Ryan K., Edelson J., Neff C., Cioffi G., Waite K.A., Kruchko C., Barnholtz-Sloan J.S. (2022). CBTRUS Statistical Report: Pediatric Brain Tumor Foundation Childhood and Adolescent Primary Brain and Other Central Nervous System Tumors Diagnosed in the United States in 2014–2018. Neuro Oncol..

[6] Stupp R., Mason W.P., van den Bent M.J., Weller M., Fisher B., Taphoorn M.J.B., Belanger K., Brandes A.A., Marosi C., Bogdahn U. (2005). Radiotherapy plus Concomitant and Adjuvant Temozolomide for Glioblastoma. N. Engl. J. Med..

[7] Stupp R., Hegi M.E., Mason W.P., van den Bent M.J., Taphoorn M.J.B., Janzer R.C., Ludwin S.K., Allgeier A., Fisher B., Belanger K. (2009). Effects of radiotherapy with concomitant and adjuvant temozolomide versus radiotherapy alone on survival in glioblastoma in a randomised phase III study: 5-year analysis of the EORTC-NCIC trial. Lancet Oncol..

[8] Kitange G.J., Carlson B.L., Schroeder M.A., Grogan P.T., Lamont J.D., Decker P.A., Wu W., James C.D., Sarkaria J.N. (2009). Induction of MGMT expression is associated with temozolomide resistance in glioblastoma xenografts. Neuro Oncol..

[9] Gilbert M.R., Dignam J.J., Armstrong T.S., Wefel J.S., Blumenthal D.T., Vogelbaum M.A., Colman H., Chakravarti A., Pugh S., Won M. (2014). A Randomized Trial of Bevacizumab for Newly Diagnosed Glioblastoma. N. Engl. J. Med..

[10] Le Rhun E., Preusser M., Roth P., Reardon D.A., van den Bent M., Wen P., Reifenberger G., Weller M. (2019). Molecular targeted therapy of glioblastoma. Cancer Treat Rev..

[11] Collins V.P. (2007). Mechanisms of disease: Genetic predictors of response to treatment in brain tumors. Nat. Clin. Pract. Oncol..

[12] Bieging K.T., Mello S.S., Attardi L.D. (2014). Unravelling mechanisms of p53-mediated tumour suppression. Nature reviews. Cancer.

[13] Kastenhuber E.R., Lowe S.W. (2017). Putting p53 in Context. Cell.

[14] Ventura A., Kirsch D.G., McLaughlin M.E., Tuveson D.A., Grimm J., Lintault L., Newman J., Reczek E.E., Weissleder R., Jacks T. (2007). Restoration of p53 function leads to tumour regression in vivo. Nature.

[15] Feldser D.M., Kostova K.K., Winslow M.M., Taylor S.E., Cashman C., Whittaker C.A., Sanchez-Rivera F.J., Resnick R., Bronson R., Hemann M.T. (2010). Stage-specific sensitivity to p53 restoration during lung cancer progression. Nature.

[16] Dickins R.A., Hemann M.T., Zilfou J.T., Simpson D.R., Ibarra I., Hannon G.J., Lowe S.W. (2006). Probing Tumor Phenotypes Using Stable and Regulated Synthetic microRNA Precursors. Nat. Genet..

[17] Stewart-Ornstein J., Lahav G. (2017). p53 dynamics in response to DNA damage vary across cell lines and are shaped by efficiency of DNA repair and activity of the kinase ATM. Sci. Signal..

[18] Jiang L., Sheikh M.S., Huang Y. (2010). Decision Making by p53: Life versus Death. Mol. Cell. Pharmacol..

[19] Wu L., Zhou N., Sun R., Chen X.D., Feng S.C., Zhang B., Bao J.K. (2014). Network-based identification of key proteins involved in apoptosis and cell cycle regulation. Cell Prolif..

[20] Wu M., Ye H., Tang Z., Shao C., Lu G., Chen B., Yang Y., Wang G., Hao H. (2017). p53 dynamics orchestrates with binding affinity to target genes for cell fate decision. Cell Death Dis..

[21] Wang P., Guan D., Zhang X.P., Liu F., Wang W. (2019). Modeling the regulation of p53 activation by HIF-1 upon hypoxia. FEBS Lett..

[22] Fischer M., Grossmann P., Padi M., DeCaprio J.A. (2016). Integration of TP53, DREAM, MMB-FOXM1 and RB-E2F target gene analyses identifies cell cycle gene regulatory networks. Nucleic Acids Res..

[23] Vogelstein B., Lane D., Levine A.J. (2000). Surfing the p53 network. Nature.

[24] Joerger A.C., Fersht A.R. (2016). The p53 Pathway: Origins, Inactivation in Cancer, and Emerging Therapeutic Approaches. Annu. Rev. Biochem..

[25] Vassilev L.T. (2007). MDM2 inhibitors for cancer therapy. Trends Mol. Med..

[26] Levine A.J., Oren M. (2009). The first 30 years of p53: Growing ever more complex. Nature reviews. Cancer.

[27] Sabapathy K., Lane D.P. (2018). Therapeutic targeting of p53: All mutants are equal, but some mutants are more equal than others. Nat. Rev. Clin. Oncol..

[28] Network C.G.A.R. (2008). Comprehensive genomic characterization defines human glioblastoma genes and core pathways. Nature.

[29] Liu J., Lichtenberg T., Hoadley K.A., Poisson L.M., Lazar A.J., Cherniack A.D., Kovatich A.J., Benz C.C., Levine D.A., Lee A.V. (2018). An Integrated TCGA Pan-Cancer Clinical Data Resource to Drive High-Quality Survival Outcome Analytics. Cell.

[30] Guo C.F., Zhuang Y., Chen Y., Chen S., Peng H., Zhou S. (2020). Significance of tumor protein p53 mutation in cellular process and drug selection in brain lower grade (WHO grades II and III) glioma. Biomark Med..

[31] Noor H., Briggs N.E., McDonald K.L., Holst J., Vittorio O. (2021). TP53 Mutation Is a Prognostic Factor in Lower Grade Glioma and May Influence Chemotherapy Efficacy. Cancers.

[32] Marker D.F., Agnihotri S., Amankulor N., Murdoch G.H., Pearce T.M. (2022). The dominant TP53 hotspot mutation in IDH -mutant astrocytoma, R273C, has distinctive pathologic features and sex-specific prognostic implications. Neurooncol. Adv..

[33] Cerami E., Gao J., Dogrusoz U., Gross B.E., Sumer S.O., Aksoy B.A., Jacobsen A., Byrne C.J., Heuer M.L., Larsson E. (2012). The cBio cancer genomics portal: An open platform for exploring multidimensional cancer genomics data. Cancer Discov..

[34] Gao J., Aksoy B.A., Dogrusoz U., Dresdner G., Gross B., Sumer S.O., Sun Y., Jacobsen A., Sinha R., Larsson E. (2013). Integrative analysis of complex cancer genomics and clinical profiles using the cBioPortal. Sci. Signal..

[35] Brennan C.W., Verhaak R.G., McKenna A., Campos B., Noushmehr H., Salama S.R., Zheng S., Chakravarty D., Sanborn J.Z., Berman S.H. (2013). The somatic genomic landscape of glioblastoma. Cell.

[36] Liu Y., Chen C., Xu Z., Scuoppo C., Rillahan C.D., Gao J., Spitzer B., Bosbach B., Kastenhuber E.R., Baslan T. (2016). Deletions linked to TP53 loss drive cancer through p53-independent mechanisms. Nature.

[37] Kato S., Han S.Y., Liu W., Otsuka K., Shibata H., Kanamaru R., Ishioka C. (2003). Understanding the function-structure and function-mutation relationships of p53 tumor suppressor protein by high-resolution missense mutation analysis. Proc. Natl. Acad. Sci. USA.

[38] Devine T., Dai M.-S. (2013). Targeting the ubiquitin-mediated proteasome degradation of p53 for cancer therapy. Curr. Pharm. Des..

[39] Carter S., Bischof O., Dejean A., Vousden K.H. (2007). C-terminal modifications regulate MDM2 dissociation and nuclear export of p53. Nat. Cell Biol..

[40] Momand J., Zambetti G.P., Olson D.C., George D., Levine A.J. (1992). The mdm-2 oncogene product forms a complex with the p53 protein and inhibits p53-mediated transactivation. Cell.

[41] Karni-Schmidt O., Lokshin M., Prives C. (2016). The Roles of MDM2 and MDMX in Cancer. Annu. Rev. Pathol..

[42] Quintás-Cardama A., Hu C., Qutub A., Qiu Y.H., Zhang X., Post S.M., Zhang N., Coombes K., Kornblau S.M. (2017). p53 pathway dysfunction is highly prevalent in acute myeloid leukemia independent of TP53 mutational status. Leukemia.

[43] Momand J., Jung D., Wilczynski S., Niland J. (1998). The MDM2 gene amplification database. Nucleic Acids Res..

[44] Shangary S., Wang S. (2009). Small-molecule inhibitors of the MDM2-p53 protein-protein interaction to reactivate p53 function: A novel approach for cancer therapy. Annu. Rev. Pharmacol. Toxicol..

[45] Kato S., Ross J.S., Gay L., Dayyani F., Roszik J., Subbiah V., Kurzrock R. (2018). Analysis of MDM2 Amplification: Next-Generation Sequencing of Patients with Diverse Malignancies. JCO Precis Oncol..

[46] Dentro S.C., Leshchiner I., Haase K., Tarabichi M., Wintersinger J., Deshwar A.G., Yu K., Rubanova Y., Macintyre G., Demeulemeester J. (2021). Characterizing genetic intra-tumor heterogeneity across 2658 human cancer genomes. Cell.

[47] Konopleva M., Martinelli G., Daver N., Papayannidis C., Wei A., Higgins B., Ott M., Mascarenhas J., Andreeff M. (2020). MDM2 inhibition: An important step forward in cancer therapy. Leukemia.

[48] Zhang B., Golding B.T., Hardcastle I.R. (2015). Small-molecule MDM2-p53 inhibitors: Recent advances. Future Med. Chem..

[49] Zhao Y., Aguilar A., Bernard D., Wang S. (2015). Small-molecule inhibitors of the MDM2-p53 protein-protein interaction (MDM2 Inhibitors) in clinical trials for cancer treatment. J. Med. Chem..

[50] Wang S., Zhao Y., Aguilar A., Bernard D., Yang C.Y. (2017). Targeting the MDM2-p53 Protein-Protein Interaction for New Cancer Therapy: Progress and Challenges. Cold Spring Harb. Perspect. Med..

[51] Lakoma A., Barbieri E., Agarwal S., Jackson J., Chen Z., Kim Y., McVay M., Shohet J.M., Kim E.S. (2015). The MDM2 small-molecule inhibitor RG7388 leads to potent tumor inhibition in p53 wild-type neuroblastoma. Cell Death Discov..

[52] Verreault M., Schmitt C., Goldwirt L., Pelton K., Haidar S., Levasseur C., Guehennec J., Knoff D., Labussiere M., Marie Y. (2016). Preclinical Efficacy of the MDM2 Inhibitor RG7112 in MDM2-Amplified and TP53 Wild-type Glioblastomas. Clin. Cancer Res..

[53] Burgess A., Chia K.M., Haupt S., Thomas D., Haupt Y., Lim E. (2016). Clinical Overview of MDM2/X-Targeted Therapies. Front. Oncol..

[54] Tisato V., Voltan R., Gonelli A., Secchiero P., Zauli G. (2017). MDM2/X inhibitors under clinical evaluation: Perspectives for the management of hematological malignancies and pediatric cancer. J. Hematol. Oncol..

[55] Duffy M.J., Synnott N.C., O’Grady S., Crown J. (2022). Targeting p53 for the treatment of cancer. Semin. Cancer Biol..

[56] Vassilev L.T., Vu B.T., Graves B., Carvajal D., Podlaski F., Filipovic Z., Kong N., Kammlott U., Lukacs C., Klein C. (2004). In vivo activation of the p53 pathway by small-molecule antagonists of MDM2. Science.

[57] Tovar C., Rosinski J., Filipovic Z., Higgins B., Kolinsky K., Hilton H., Zhao X., Vu B.T., Qing W., Packman K. (2006). Small-molecule MDM2 antagonists reveal aberrant p53 signaling in cancer: Implications for therapy. Proc. Natl. Acad. Sci. USA.

[58] Ding Q., Zhang Z., Liu J.J., Jiang N., Zhang J., Ross T.M., Chu X.J., Bartkovitz D., Podlaski F., Janson C. (2013). Discovery of RG7388, a potent and selective p53-MDM2 inhibitor in clinical development. J. Med. Chem..

[59] Kojima K., Konopleva M., Samudio I.J., Shikami M., Cabreira-Hansen M., McQueen T., Ruvolo V., Tsao T., Zeng Z., Vassilev L.T. (2005). MDM2 antagonists induce p53-dependent apoptosis in AML: Implications for leukemia therapy. Blood.

[60] Villalonga-Planells R., Coll-Mulet L., Martínez-Soler F., Castaño E., Acebes J.J., Giménez-Bonafé P., Gil J., Tortosa A. (2011). Activation of p53 by nutlin-3a induces apoptosis and cellular senescence in human glioblastoma multiforme. PLoS ONE.

[61] Vu B., Wovkulich P., Pizzolato G., Lovey A., Ding Q., Jiang N., Liu J.J., Zhao C., Glenn K., Wen Y. (2013). Discovery of RG7112: A Small-Molecule MDM2 Inhibitor in Clinical Development. ACS Med. Chem. Lett..

[62] Lu M., Wang X., Li Y., Tripodi J., Mosoyan G., Mascarenhas J., Kremyanskaya M., Najfeld V., Hoffman R. (2012). Combination treatment in vitro with Nutlin, a small-molecule antagonist of MDM2, and pegylated interferon-α 2a specifically targets JAK2V617F-positive polycythemia vera cells. Blood.

[63] Lu M., Xia L., Li Y., Wang X., Hoffman R. (2014). The orally bioavailable MDM2 antagonist RG7112 and pegylated interferon α 2a target JAK2V617F-positive progenitor and stem cells. Blood.

[64] Ray-Coquard I., Blay J.Y., Italiano A., Le Cesne A., Penel N., Zhi J., Heil F., Rueger R., Graves B., Ding M. (2012). Effect of the MDM2 antagonist RG7112 on the P53 pathway in patients with MDM2-amplified, well-differentiated or dedifferentiated liposarcoma: An exploratory proof-of-mechanism study. Lancet Oncol..

[65] Andreeff M., Kelly K.R., Yee K., Assouline S., Strair R., Popplewell L., Bowen D., Martinelli G., Drummond M.W., Vyas P. (2016). Results of the Phase I Trial of RG7112, a Small-Molecule MDM2 Antagonist in Leukemia. Clin. Cancer Res..

[66] Higgins B., Tovar C., Glenn K., Walz A., Filipovic Z., Zhang Y.-E., Dangl M., Graves B., Vassilev L., Packman K. (2013). Abstract B55: Antitumor activity of the MDM2 antagonist RG7388. Mol. Cancer Ther..

[67] Berberich A., Kessler T., Thomé C.M., Pusch S., Hielscher T., Sahm F., Oezen I., Schmitt L.M., Ciprut S., Hucke N. (2019). Targeting Resistance against the MDM2 Inhibitor RG7388 in Glioblastoma Cells by the MEK Inhibitor Trametinib. Clin. Cancer Res..

[68] Siu L.L., Italiano A., Miller W.H., Blay J.-Y., Gietema J.A., Bang Y.-J., Mileshkin L.R., Hirte H.W., Reckner M., Higgins B. (2014). Phase 1 dose escalation, food effect, and biomarker study of RG7388, a more potent second-generation MDM2 antagonist, in patients (pts) with solid tumors. J. Clin. Oncol..

[69] Higgins B., Glenn K., Walz A., Tovar C., Filipovic Z., Hussain S., Lee E., Kolinsky K., Tannu S., Adames V. (2014). Preclinical optimization of MDM2 antagonist scheduling for cancer treatment by using a model-based approach. Clin. Cancer Res..

[70] Sun D., Li Z., Rew Y., Gribble M., Bartberger M.D., Beck H.P., Canon J., Chen A., Chen X., Chow D. (2014). Discovery of AMG 232, a potent, selective, and orally bioavailable MDM2-p53 inhibitor in clinical development. J. Med. Chem..

[71] Canon J., Osgood T., Olson S.H., Saiki A.Y., Robertson R., Yu D., Eksterowicz J., Ye Q., Jin L., Chen A. (2015). The MDM2 Inhibitor AMG 232 Demonstrates Robust Antitumor Efficacy and Potentiates the Activity of p53-Inducing Cytotoxic Agents. Mol. Cancer Ther..

[72] Her N.G., Oh J.W., Oh Y.J., Han S., Cho H.J., Lee Y., Ryu G.H., Nam D.H. (2018). Potent effect of the MDM2 inhibitor AMG232 on suppression of glioblastoma stem cells. Cell Death Dis..

[73] Wang S., Sun W., Zhao Y., McEachern D., Meaux I., Barrière C., Stuckey J.A., Meagher J.L., Bai L., Liu L. (2014). SAR405838: An optimized inhibitor of MDM2-p53 interaction that induces complete and durable tumor regression. Cancer Res..

[74] Kim M., Ma D.J., Calligaris D., Zhang S., Feathers R.W., Vaubel R.A., Meaux I., Mladek A.C., Parrish K.E., Jin F. (2018). Efficacy of the MDM2 Inhibitor SAR405838 in Glioblastoma Is Limited by Poor Distribution Across the Blood-Brain Barrier. Mol. Cancer Ther..

[75] de Jonge M., de Weger V.A., Dickson M.A., Langenberg M., Le Cesne A., Wagner A.J., Hsu K., Zheng W., Macé S., Tuffal G. (2017). A phase I study of SAR405838, a novel human double minute 2 (HDM2) antagonist, in patients with solid tumours. Eur. J. Cancer.

[76] de Weger V.A., de Jonge M., Langenberg M.H.G., Schellens J.H.M., Lolkema M., Varga A., Demers B., Thomas K., Hsu K., Tuffal G. (2019). A phase I study of the HDM2 antagonist SAR405838 combined with the MEK inhibitor pimasertib in patients with advanced solid tumours. Br. J. Cancer.

[77] Aguilar A., Lu J., Liu L., Du D., Bernard D., McEachern D., Przybranowski S., Li X., Luo R., Wen B. (2017). Discovery of 4-((3′ R, 4′ S, 5′ R)-6 ″-Chloro-4′-(3-chloro-2-fluorophenyl)-1′-ethyl-2 ″-oxodispiro [cyclohexane-1, 2′-pyrrolidine-3′, 3 ″-indoline]-5′-carboxamido) bicyclo [2.2.2] octane-1-carboxylic Acid (AA-115/APG-115): A Potent and Orally Active Murine Double Minute 2 (MDM2) Inhibitor in Clinical Development. J. Med. Chem..

[78] Skalniak L., Surmiak E., Holak T.A. (2019). A therapeutic patent overview of MDM2/X-targeted therapies (2014-2018). Expert Opin. Ther. Pat..

[79] Chong C.R., Bauer T.M., Laurie S.A., Patel M.R., Yamamoto N., Davenport T., Geng J., Gibson N., Vallaster M.P., LoRusso P. (2019). A phase Ia/Ib, open label, multicenter, dose-escalation study of BI 907828 (MDM2-p53 antagonist) in adult patients with advanced or metastatic solid tumors. J. Clin. Oncol..

[80] Hao X., Bahia R., Cseh O., Bozek D., Blake S., Rinnenthal J., Weyer-Czernilofsky U., Rudolph D., Luchman H.A. (2023). BI-907828, a novel potent MDM2 inhibitor, inhibits GBM brain tumor stem cells in vitro and prolongs survival in orthotopic xenograft mouse models. Neuro-Oncol..

[81] Cornillie J., Wozniak A., Li H., Gebreyohannes Y.K., Wellens J., Hompes D., Debiec-Rychter M., Sciot R., Schoffski P. (2020). Anti-tumor activity of the MDM2-TP53 inhibitor BI-907828 in dedifferentiated liposarcoma patient-derived xenograft models harboring MDM2 amplification. Clin. Transl. Oncol..

[82] Rinnenthal J., Rudolph D., Blake S., Gollner A., Wernitznig A., Weyer-Czernilofsky U., Haslinger C., Garin-Chesa P., Moll J., Kraut N. (2018). BI 907828: A highly potent MDM2 inhibitor with low human dose estimation, designed for high-dose intermittent schedules in the clinic. Cancer Res..

[83] Gessier F., Kallen J., Jacoby E., Chène P., Stachyra-Valat T., Ruetz S., Jeay S., Holzer P., Masuya K., Furet P. (2015). Discovery of dihydroisoquinolinone derivatives as novel inhibitors of the p53-MDM2 interaction with a distinct binding mode. Bioorg. Med. Chem. Lett..

[84] Holzer P., Masuya K., Furet P., Kallen J., Valat-Stachyra T., Ferretti S., Berghausen J., Bouisset-Leonard M., Buschmann N., Pissot-Soldermann C. (2015). Discovery of a Dihydroisoquinolinone Derivative (NVP-CGM097): A Highly Potent and Selective MDM2 Inhibitor Undergoing Phase 1 Clinical Trials in p53wt Tumors. J. Med. Chem..

[85] Zhang M., Chen X.Y., Dong X.D., Wang J.Q., Feng W., Teng Q.X., Cui Q., Li J., Li X.Q., Chen Z.S. (2020). NVP-CGM097, an HDM2 Inhibitor, Antagonizes ATP-Binding Cassette Subfamily B Member 1-Mediated Drug Resistance. Front. Oncol..

[86] Maser T., Zagorski J., Kelly S., Ostrander A., Goodyke A., Nagulapally A., Bond J., Park Y., Saulnier Sholler G. (2020). The MDM2 inhibitor CGM097 combined with the BET inhibitor OTX015 induces cell death and inhibits tumor growth in models of neuroblastoma. Cancer Med..

[87] Reuther C., Heinzle V., Nölting S., Herterich S., Hahner S., Halilovic E., Jeay S., Wuerthner J.U., Aristizabal Prada E.T., Spöttl G. (2018). The HDM2 (MDM2) Inhibitor NVP-CGM097 Inhibits Tumor Cell Proliferation and Shows Additive Effects with 5-Fluorouracil on the p53-p21-Rb-E2F1 Cascade in the p53wild type Neuroendocrine Tumor Cell Line GOT1. Neuroendocrinology.

[88] Townsend E.C., DeSouza T., Murakami M.A., Montero J., Stevenson K., Christie A.L., Christodolou A.N., Vojinovic U., Kopp N., Barzaghi-Rinaudo P. (2015). The MDM2 inhibitor NVP-CGM097 is highly active in a randomized preclinical trial of B-cell acute lymphoblastic leukemia patient derived xenografts. Blood.

[89] Holzer P. (2017). Discovery of Potent and Selective p53-MDM2 Protein-Protein Interaction Inhibitors as Anticancer Drugs. Chimia.

[90] Ferretti S., Rebmann R., Berger M., Santacroce F., Albrecht G., Pollehn K., Sterker D., Wartmann M., Hueber A., Wiesmann M. (2016). Insights into the mechanism of action of NVP-HDM201, a differentiated and versatile Next-Generation small-molecule inhibitor of Mdm2, under evaluation in phase I clinical trials. Cancer Res..

[91] Jeay S., Chène P., Ferretti S., Furet P., Gruenenfelder B., Guagnano V., Guerreiro N., Halilovic E., Hofmann F., Kallen J. (2016). *NVP-HDM*201: Cellular and in vivo profile of a novel highly potent and selective PPI inhibitor of p53-Mdm2. Cancer Res..

[92] Jeay S., Ferretti S., Holzer P., Fuchs J., Chapeau E.A., Wartmann M., Sterker D., Romanet V., Murakami M., Kerr G. (2018). Dose and Schedule Determine Distinct Molecular Mechanisms Underlying the Efficacy of the p53-MDM2 Inhibitor HDM201. Cancer Res..

[93] Vaupel A., Holzer P., Ferretti S., Guagnano V., Kallen J., Mah R., Masuya K., Ruetz S., Rynn C., Schlapbach A. (2018). In vitro and in vivo characterization of a novel, highly potent p53-MDM2 inhibitor. Bioorg. Med. Chem. Lett..

[94] Stein E., Chromik J., DeAngelo D.J., Chatterjee M., Noppeney R., Vos F.d., Minami H., Jeay S., Meille C., Halilovic E. (2017). *Abstract CT*152: Phase I dose-and regimen-finding study of NVP-HDM201 in pts with advanced TP53 wt acute leukemias. Cancer Res..

[95] Hyman D.M., Chatterjee M., de Vos F., Lin C.-C., Suarez C., Tai D., Cassier P., Yamamoto N., de Weger V.A., Jeay S. Optimizing the therapeutic index of HDM2 inhibition: Results from a dose-and regimen-finding phase I study of NVP-HDM201 in pts with TP53 wt advanced tumors. Proceedings of the Cancer Research.

[96] Arnhold V., Schmelz K., Proba J., Winkler A., Wünschel J., Toedling J., Deubzer H.E., Künkele A., Eggert A., Schulte J.H. (2018). Reactivating TP53 signaling by the novel MDM2 inhibitor DS-3032b as a therapeutic option for high-risk neuroblastoma. Oncotarget.

[97] da Mota V.H.S., Freire de Melo F., de Brito B.B., da Silva F.A.F., Teixeira K.N. (2022). Molecular docking of DS-3032B, a mouse double minute 2 enzyme antagonist with potential for oncology treatment development. World J. Clin. Oncol..

[98] Ishizawa J., Nakamaru K., Seki T., Tazaki K., Kojima K., Chachad D., Zhao R., Heese L., Ma W., Ma M.C.J. (2018). Predictive Gene Signatures Determine Tumor Sensitivity to MDM2 Inhibition. Cancer Res..

[99] Ananthapadmanabhan V., Frost T.C., Soroko K.M., Knott A., Magliozzi B.J., Gokhale P.C., Tirunagaru V.G., Doebele R.C., DeCaprio J.A. (2022). Milademetan is a highly potent MDM2 inhibitor in Merkel cell carcinoma. JCI Insight.

[100] Takahashi S., Fujiwara Y., Nakano K., Shimizu T., Tomomatsu J., Koyama T., Ogura M., Tachibana M., Kakurai Y., Yamashita T. (2021). Safety and pharmacokinetics of milademetan, a MDM2 inhibitor, in Japanese patients with solid tumors: A phase I study. Cancer Sci..

[101] Sekiguchi N., Kasahara S., Miyamoto T., Kiguchi T., Ohno H., Takagi T., Tachibana M., Sumi H., Kakurai Y., Yamashita T. (2023). Phase I dose-escalation study of milademetan in patients with relapsed or refractory acute myeloid leukemia. Int. J. Hematol..

[102] Gounder M.M., Bauer T.M., Schwartz G.K., Weise A.M., LoRusso P., Kumar P., Tao B., Hong Y., Patel P., Lu Y. (2023). A First-in-Human Phase I Study of Milademetan, an MDM2 Inhibitor, in Patients with Advanced Liposarcoma, Solid Tumors, or Lymphomas. J. Clin. Oncol..

[103] Golestanian S., Sharifi A., Popowicz G.M., Azizian H., Foroumadi A., Szwagierczak A., Holak T.A., Amanlou M. (2016). Discovery of novel dual inhibitors against Mdm2 and Mdmx proteins by in silico approaches and binding assay. Life Sci..

[104] Lemos A., Leão M., Soares J., Palmeira A., Pinto M., Saraiva L., Sousa M.E. (2016). Medicinal Chemistry Strategies to Disrupt the p53-MDM2/MDMX Interaction. Med. Res. Rev..

[105] Saleh M.N., Patel M.R., Bauer T.M., Goel S., Falchook G.S., Shapiro G.I., Chung K.Y., Infante J.R., Conry R.M., Rabinowits G. (2021). Phase 1 Trial of ALRN-6924, a Dual Inhibitor of MDMX and MDM2, in Patients with Solid Tumors and Lymphomas Bearing Wild-type TP53. Clin. Cancer Res..

[106] Carvajal L.A., Neriah D.B., Senecal A., Benard L., Thiruthuvanathan V., Yatsenko T., Narayanagari S.R., Wheat J.C., Todorova T.I., Mitchell K. (2018). Dual inhibition of MDMX and MDM2 as a therapeutic strategy in leukemia. Sci. Transl. Med..

[107] Pairawan S., Zhao M., Yuca E., Annis A., Evans K., Sutton D., Carvajal L., Ren J.G., Santiago S., Guerlavais V. (2021). First in class dual MDM2/MDMX inhibitor ALRN-6924 enhances antitumor efficacy of chemotherapy in TP53 wild-type hormone receptor-positive breast cancer models. Breast Cancer Res..

[108] Fisher J.P., Adamson D.C. (2021). Current FDA-Approved Therapies for High-Grade Malignant Gliomas. Biomedicines.

[109] Fernandes C., Costa A., Osório L., Lago R.C., Linhares P., Carvalho B., Caeiro C. (2017). Current Standards of Care in Glioblastoma Therapy. Glioblastoma.

[110] Nickoloff J.A., Taylor L., Sharma N., Kato T.A. (2021). Exploiting DNA repair pathways for tumor sensitization, mitigation of resistance, and normal tissue protection in radiotherapy. Cancer Drug Resist..

[111] Toulany M. (2019). Targeting DNA Double-Strand Break Repair Pathways to Improve Radiotherapy Response. Genes.

[112] Liu Y.P., Zheng C.C., Huang Y.N., He M.L., Xu W.W., Li B. (2021). Molecular mechanisms of chemo- and radiotherapy resistance and the potential implications for cancer treatment. MedComm.

[113] Hakem R. (2008). DNA-damage repair; the good, the bad, and the ugly. EMBO J..

[114] Helleday T., Petermann E., Lundin C., Hodgson B., Sharma R.A. (2008). DNA repair pathways as targets for cancer therapy. Nat. Rev. Cancer.

[115] Aubrey B.J., Kelly G.L., Janic A., Herold M.J., Strasser A. (2018). How does p53 induce apoptosis and how does this relate to p53-mediated tumour suppression?. Cell Death Differ..

[116] O’Brien M.A., Kirby R. (2008). Apoptosis: A review of pro-apoptotic and anti-apoptotic pathways and dysregulation in disease. J. Vet. Emerg. Crit. Care.

[117] Bagnyukova T.V., Serebriiskii I.G., Zhou Y., Hopper-Borge E.A., Golemis E.A., Astsaturov I. (2010). Chemotherapy and signaling. Cancer Biol. Ther..

[118] Sun Y., Liu Y., Ma X., Hu H. (2021). The Influence of Cell Cycle Regulation on Chemotherapy. Int. J. Mol. Sci..

[119] Woods D., Turchi J.J. (2013). Chemotherapy induced DNA damage response. Cancer Biol. Ther..

[120] Van Den Boogaard W.M.C., Komninos D.S.J., Vermeij W.P. (2022). Chemotherapy Side-Effects: Not All DNA Damage Is Equal. Cancers.

[121] Swift L., Golsteyn R. (2014). Genotoxic Anti-Cancer Agents and Their Relationship to DNA Damage, Mitosis, and Checkpoint Adaptation in Proliferating Cancer Cells. Int. J. Mol. Sci..

[122] Shangary S., Wang S. (2008). Targeting the MDM2-p53 Interaction for Cancer Therapy. Clin. Cancer Res. Off. J. Am. Assoc. Cancer Res..

[123] Shen H., G Maki C. (2011). Pharmacologic Activation of p53 by Small-Molecule MDM2 Antagonists. Curr. Pharm. Des..

[124] Nag S., Zhang X., Srivenugopal K.S., Wang M.H., Wang W., Zhang R. (2014). Targeting MDM2-p53 Interaction for Cancer Therapy: Are We There Yet?. Curr. Med. Chem..

[125] Zhu H., Gao H., Ji Y., Zhou Q., Du Z., Tian L., Jiang Y., Yao K., Zhou Z. (2022). Targeting p53–MDM2 interaction by small-molecule inhibitors: Learning from MDM2 inhibitors in clinical trials. J. Hematol. Oncol..

[126] Carr M.I., Jones S.N. (2016). Regulation of the Mdm2-p53 signaling axis in the DNA damage response and tumorigenesis. Transl. Cancer Res..

[127] Klein A.M., De Queiroz R.M., Venkatesh D., Prives C. (2021). The roles and regulation of MDM2 and MDMX: It is not just about p53. Genes Dev..

[128] Chen J. (2016). The Cell-Cycle Arrest and Apoptotic Functions of p53 in Tumor Initiation and Progression. Cold Spring Harb. Perspect. Med..

[129] Ozaki T., Nakagawara A. (2011). Role of p53 in Cell Death and Human Cancers. Cancers.

[130] Feroz W., Sheikh A.M.A. (2020). Exploring the multiple roles of guardian of the genome: P53. Egypt. J. Med. Hum. Genet..

[131] Miles X., Vandevoorde C., Hunter A., Bolcaen J. (2021). MDM2/X Inhibitors as Radiosensitizers for Glioblastoma Targeted Therapy. Front. Oncol..

[132] Welliver M.X., Tine B.A.V., Houghton P., Rudek M.A., Pollock R.E., Kane J.M., Schwartz G.K., Zhang P., Kirsch D.G., Wakely P. (2019). MDM2 inhibitor AMG-232 and radiation therapy in treating patients with soft tissue sarcoma with wild-type TP53: A phase IB study (NRG-DT001). J. Clin. Oncol..

[133] Me P. (2004). Mdm2 in the response to radiation. Mol. Cancer Res. MCR.

[134] Geske F.J., Nelson A.C., Lieberman R., Strange R., Sun T., Gerschenson L.E. (2000). DNA repair is activated in early stages of p53-induced apoptosis. Cell Death Differ..

[135] Koo N., Sharma A.K., Narayan S. (2022). Therapeutics Targeting p53-MDM2 Interaction to Induce Cancer Cell Death. Int. J. Mol. Sci..

[136] Prabakaran P.J., Javaid A.M., Swick A.D., Werner L.R., Nickel K.P., Sampene E., Hu R., Ong I.M., Bruce J.Y., Hartig G.K. (2017). Radiosensitization of Adenoid Cystic Carcinoma with MDM2 Inhibition. Clin. Cancer Res..

[137] Luo H., Yount C., Lang H., Yang A., Riemer E.C., Lyons K., Vanek K.N., Silvestri G.A., Schulte B.A., Wang G.Y. (2013). Activation of p53 with Nutlin-3a radiosensitizes lung cancer cells via enhancing radiation-induced premature senescence. Lung Cancer.

[138] Mladek A.C., Gupta S., Kim M., Mohammad A.S., Bakken K.K., He H., Hu Z., Burgenske D.M., Carlson B.L., Elmquist W.F. (2019). Abstract C051: MDM2 inhibitor KRT-232 extends survival in glioblastoma patient-derived xenograft models. Mol. Cancer Ther..

[139] Eischen C.M. (2023). Role of Mdm2 and Mdmx in DNA repair. J. Mol. Cell Biol..

[140] Hientz K., Mohr A., Bhakta-Guha D., Efferth T. (2017). The role of p53 in cancer drug resistance and targeted chemotherapy. Oncotarget.

[141] Chen L., Agrawal S., Zhou W., Zhang R., Chen J. (1998). Synergistic activation of p53 by inhibition of MDM2 expression and DNA damage. Proc. Natl. Acad. Sci. USA.

[142] Saiki A.Y., Caenepeel S., Yu D., Lofgren J.A., Osgood T., Robertson R., Canon J., Su C., Jones A., Zhao X. (2014). MDM2 antagonists synergize broadly and robustly with compounds targeting fundamental oncogenic signaling pathways. Oncotarget.

[143] Sarisozen C., Tan Y., Liu J., Bilir C., Shen L., Filipczak N., Porter T.M., Torchilin V.P. (2019). MDM2 antagonist-loaded targeted micelles in combination with doxorubicin: Effective synergism against human glioblastoma via p53 re-activation. J. Drug Target..

[144] Tong H., Zhao K., Zhang J., Zhu J., Xiao J. (2019). YB-1 modulates the drug resistance of glioma cells by activation of MDM2/p53 pathway. Drug Des. Dev. Ther..

[145] Chen X., Tai L., Gao J., Qian J., Zhang M., Li B., Xie C., Lu L., Lu W., Lu W. (2015). A stapled peptide antagonist of MDM2 carried by polymeric micelles sensitizes glioblastoma to temozolomide treatment through p53 activation. J. Control. Release.

[146] Costa B., Bendinelli S., Gabelloni P., Da Pozzo E., Daniele S., Scatena F., Vanacore R., Campiglia P., Bertamino A., Gomez-Monterrey I. (2013). Human Glioblastoma Multiforme: p53 Reactivation by a Novel MDM2 Inhibitor. PLoS ONE.

[147] Wang H., Cai S., Bailey B.J., Reza Saadatzadeh M., Ding J., Tonsing-Carter E., Georgiadis T.M., Zachary Gunter T., Long E.C., Minto R.E. (2017). Combination therapy in a xenograft model of glioblastoma: Enhancement of the antitumor activity of temozolomide by an MDM2 antagonist. J. Neurosurg. JNS.

[148] Pi L., Rooprai J., Allan D.S., Atkins H., Bredeson C., Fulcher A.J., Ito C., Ramsay T., Stanford W.L., Sabloff M. (2019). Evaluating dose-limiting toxicities of MDM2 inhibitors in patients with solid organ and hematologic malignancies: A systematic review of the literature. Leuk. Res..

[149] Sato A., Sunayama J., Matsuda K.-I., Seino S., Suzuki K., Watanabe E., Tachibana K., Tomiyama A., Kayama T., Kitanaka C. (2011). MEK-ERK signaling dictates DNA-repair gene MGMT expression and temozolomide resistance of stem-like glioblastoma cells via the MDM2-p53 axis. Stem Cells.

[150] Felsberg J., Hentschel B., Kaulich K., Gramatzki D., Zacher A., Malzkorn B., Kamp M., Sabel M., Simon M., Westphal M. (2017). Epidermal Growth Factor Receptor Variant III (EGFRvIII) Positivity in EGFR-Amplified Glioblastomas: Prognostic Role and Comparison between Primary and Recurrent Tumors. Clin. Cancer Res..

[151] Daniele S., Costa B., Zappelli E., Da Pozzo E., Sestito S., Nesi G., Campiglia P., Marinelli L., Novellino E., Rapposelli S. (2015). Combined inhibition of AKT/mTOR and MDM2 enhances Glioblastoma Multiforme cell apoptosis and differentiation of cancer stem cells. Sci. Rep..

[152] (2023). A Phase 1 Study Evaluating AMG 232 in Advanced Solid Tumors or Multiple Myeloma—Full Text View. https://ClinicalTrials.gov.

[153] (2023). 1Testing the Ability of AMG 232 (KRT 232) to Get into the Tumor in Patients with Brain Cancer—Full Text View. https://ClinicalTrials.gov.

[154] (2023). NCT Neuro Master Match-N²M² (NOA-20)—Full Text View. https://ClinicalTrials.gov.

[155] (2023). Phase 1 Study of the Dual MDM2/MDMX Inhibitor ALRN-6924 in Pediatric Cancer—Full Text View. https://ClinicalTrials.gov.

[156] (2023). A Study to Determine How BI 907828 is Taken up in the Tumor and to Determine the Highest Dose of BI 907828 that Could be Tolerated in Combination with Radiation Therapy in People with a Brain Tumor Called Glioblastoma—Full Text View. https://ClinicalTrials.gov.

[157] Schöffski P., Lahmar M., Lucarelli A., Maki R.G. (2023). Brightline-1: Phase II/III trial of the MDM2-p53 antagonist BI 907828 versus doxorubicin in patients with advanced DDLPS. Future Oncol..

[158] Montesinos P., Beckermann B.M., Catalani O., Esteve J., Gamel K., Konopleva M.Y., Martinelli G., Monnet A., Papayannidis C., Park A. (2020). MIRROS: A randomized, placebo-controlled, Phase III trial of cytarabine ± idasanutlin in relapsed or refractory acute myeloid leukemia. Future Oncol..

[159] Konopleva M.Y., Röllig C., Cavenagh J., Deeren D., Girshova L., Krauter J., Martinelli G., Montesinos P., Schäfer J.A., Ottmann O. (2022). Idasanutlin plus cytarabine in relapsed or refractory acute myeloid leukemia: Results of the MIRROS trial. Blood Adv..

